# Treatment of symptomatic, deep, almost cariously exposed lesions using ozone

**DOI:** 10.1038/s41598-021-90824-0

**Published:** 2021-05-27

**Authors:** Mahmoud K. AL-Omiri, Nasser M. Alqahtani, Nasser M. Alahmari, Raed Abul Hassan, Abdullah A. Al Nazeh, Edward Lynch

**Affiliations:** 1 Department of Prosthodontics, School of Dentistry, University of Jordan, Queen Rania Street, Amman, 11942 Jordan; 2 Department of Prosthodontics, The City of London Dental School, Canada Water, Lower Road, London, UK; 3 Department of Prosthodontics, College of Dentistry, King Khalid University, Asir–Abha, Saudi Arabia; 4 Faculty of Allied Medical Sciences, The Royal University for Medical Sciences, Amman, Jordan; 5 Department of Pediatric Dentistry and Orthodontics Sciences, College of Dentistry, King Khalid University, Asir–Abha, Saudi Arabia; 6 School of Dental Medicine, University of Nevada Las Vegas (UNLV), Las Vegas, NV USA

**Keywords:** Dental caries, Restorative dentistry, Pulp conservation

## Abstract

The aim of this controlled randomized crossover study was to assess post-treatment pain and the need for root canal treatment after the use of a traditional caries removal method followed by restoration, or after an ozone method of more conservatively managing the deep caries and a restoration. 84 participants (42 males and 42 females, mean age ± SD = 23.9 ± 2.0 years) were randomly allocated to receive either a traditional (n = 42, 21 males and 21 females) or ozone (n = 42, 21 males and 21 females) method. The ozone method only differed from the traditional method by leaving the deep leathery caries on the pulpal floor and then treating this with 20 s of ozone from the healozone X4 (Curozone, Germany). All caries was removed in the traditional group. A conventional glass ionomer cement (Riva Self Cure High Viscosity, SDI, Australia) was placed followed by a bonded composite resin restoration (Filtek Z250 Universal Restorative, 3 M ESPE, USA) in each cavity. The visual analogue scale was used to assess pain scores before treatment and after 24 h. The participants were then followed up for 2 years to assess the need for root canal treatment. Statistical significance levels were set at α ≤ .05. Both groups were associated with significant reduction of pain scores 24 h after treatment (p < .0001). The ozone treatment was associated with less pain 24 h after treatment (p < .0001) and less need for root canal treatment (p = .014), after 2 years follow up, than the conventional treatment. In conclusion, treatment of symptomatic, deep carious lesions by ozone following partial removal of caries was accompanied with less pain and occurrence of RCT after treatment compared to traditional complete caries removal.

## Introduction

Different treatment protocols are available to use for the management of dental caries. More interest has been instituted in management of caries using pharmaceutical agents including silver diamine fluoride and/or ozone^1–6^. This could decrease the need for conventional drilling and filling of carious lesions, and therefore might potentially enhance patients’ acceptance and compliance with the treatment^5^.

Ozone treatment is among the pharmaceutically least invasive techniques utilized for caries prevention and treatment. This paradigm might be used solely or in combination with other techniques to treat carious lesions^2,3,6–10^. Caries management using ozone is conservative, consumes less treatment time, and requires shorter periods of mouth opening^11^. In addition, ozone is efficient in killing and reducing different cariogenic microorganisms (such as streptococcus mutans, streptococcus sobrinus and lactobacilli) in both clinical and laboratory settings, especially using the healozone^1–5,8,12–31^.

Previous studies established that ozone treatment using the healozone could enhance the remineralization process and thus improve the hardness of open single surface shallow carious lesions^4,7,28^. However, significantly less cariogenic microorganisms and more arrested caries were associated with ozone application into small shallow carious lesions but not in very deep large ones^2^. It was reported that ozone application alone or in conjunction with remineralizing solutions was associated with remineralization of initial non cavitated pit, fissure, root and proximal carious lesions^2–4,6,7,9,25,32,33^. Ozone was also established to control open carious lesions in deciduous teeth^26,28,34^.

Treatment of deep carious lesions that approach the pulp is among the most important challenges in caries management. Total caries removal might lead to tooth sensitivity, pulp exposure, or the need for indirect pulp capping, direct pulp capping, pulpotomy or pulpectomy^35,36^. In an attempt to reduce these complications, conservative and ultraconservative caries management protocols including stepwise caries excavation and partial caries removal were suggested in the literature^37,38^.

However, residual bacteria might cause pulpal inflammation, secondary caries and failure of restorations. Therefore, it was suggested to perform lesion disinfection using pharmaceutical agents (such as chlorhexidine or ozone) in order to allow lesions to repair^29,39–41^. Another method was to use photodynamic antimicrobial therapy (PAD) which uses a sensitizer that is absorbed by bacteria, then the lesion is exposed to light of a specific wavelength to kill the bacteria^42^.

The literature reported no harmful consequences of applying ozone on deep caries or exposed pulps^2–4,27–29,34^. Previous studies showed that ozone was more biocompatible with human oral epithelial cells and gingival fibroblasts than chlorhexidine, sodium hypochlorite, hydrogen peroxide, and metronidazole^43^. Ozone was found to have anti-inflammatory effects on oral cells and periodontal ligament tissues from roots of periodontally involved teeth^44^. In addition, ozone was found to boost healing of traumatic and recurrent aphthous ulcers and reduce pain levels caused by ulcers^45,46^.

Indeed, the literature reported many positive effects of ozone on human tissues including oral tissues^30,44–54^. Ozone enhances the metabolism of inflamed tissues by improving blood circulation, increasing tissue oxygenation and reducing inflammation^30,44,47–49^. It stimulates immunity through proliferation of immune cells, release of endogenous interferon, synthesis of immunoglobulins, activation of macrophage function, and increasing sensitivity of microorganisms to phagocytosis^30,47,48^. It also possesses analgesic properties^30,45–47,51–53^.

However, studies on using ozone in the management of symptomatic deep caries after leaving some carious tissues before the final restoration of permanent dentition are scarce. Therefore, the current study was carried out to explore the use of ozone in management of symptomatic deep caries approaching the pulp in the permanent dentition.

The purpose of this study was to measure pain levels and the need for root canal treatment in teeth with deep carious lesions following using a conservative protocol of 20 s of ozone application on partially removed deep caries before a restoration, in comparison to a conventional treatment protocol of total caries removal and restoration.

The null hypothesis for the present study was that in symptomatic deep carious lesions approaching the pulp, 20 s of ozone application on partially removed deep caries before a restoration would lead to similar pain levels and need for root canal treatment as when a conventional treatment protocol of total caries removal and a restoration is followed.

## Materials and methods

Eighty four participants (42 males and 42 females) were recruited into this study. The study was conducted at the dental clinics, School of Dentistry, the University of Jordan (January 2017 until March 2020). Participants age ranged from 20 to 27 years old (mean age ± SD = 23.8 ± 2.0 years). This study was ethically approved by the Institutional Review Board, the University of Jordan, Amman, Jordan (Reference Number ARC-6–2015). All methods in this study were carried out in full accordance with Helsinki Declaration (9th version, 2013). This investigation was reported following the STROBE guidelines. Each potential participant was provided with a detailed explanation of the experiment, and written informed consent was obtained from each participant before inclusion in this study.

Participants were included in this experiment if they had a molar tooth with an extensive carious lesion which was causing the participants pain and which radiographically was almost cariously exposed. The involved tooth had to be vital and be sensitive to both hot and cold tests but the pain did not wake the subject up nor keep the subject awake at night. The involved tooth should not have been restored, prepared for a crown or bridge retainer, or be affected by periodontitis or extensive tooth surface loss. Participants should not have received any previous treatment for the involved carious tooth and should not have other complicating medical history. Therefore, participants who received previous treatment to the involved tooth or had their tooth affected by periodontitis or extensive tooth surface loss, or experience complicating medical history were excluded from this experiment. Participants were also excluded if they presented with temporomandibular joint problems, sinusitis, oral ulceration or any loss of lamina dura on their periapical radiograph. In addition, pregnant/lactating women were excluded from this experiment.

After recruitment, the participants were randomly distributed into two groups (n = 42 for each group). A simple randomization procedure was applied using computer generated numbers with gender stratification to avoid sex-based bias following previous recommendations^45,55^.

Each participant was thoroughly examined on a dental chair supplied with a light unit (Diamond LED Dental Light; Daray Lighting, Derbyshire, England, UK). The involved tooth was dried prior to examination. A dental mirror (15/16 inch; Hahnenkratt, Königsbach-Stein, Germany) and an explorer probe (0700–9, anatomical handle single ended; ASA Dental, Bozzano, Italy) were utilized during the intra-oral examinations. Tooth vitality was evaluated via an ice stick and compared to the response of other teeth. A pre-operative periapical digital radiograph (Ez Sensor, Vatech, Korea) was taken for the involved tooth to check for caries extension as well as to assess the periodontal and periapical conditions of the involved tooth.

Subsequently, participants’ tooth pain was assessed using a visual analogue scale (VAS) score from 0 (no tooth pain at all) to 10 (tooth pain as bad as imaginable).

A rubber dam (Sanctuary Powder Free Latex Dental Dam, 6″ × 6″ size, Sanctuary Health, Malaysia) was used with all participants during treatment of the involved teeth. In group 1, the carious lesions were treated by partial removal of caries under copious water irrigation using diamond burs (Edenta, Switzerland) mounted on contra-angled high speed handpiece (Coxo CX207-B, COXO Medical Instrument Co., China) and finally tungsten carbide burs mounted on a slow speed handpiece (Gold-U-CA, AppleDental, China). About 1 mm thickness of the deep leathery caries was left on the pulpal floor depending on measurements from preoperative periapical digital radiograph. Also, it was required that the remaining caries was leathery as determined by having resistance to withdrawal after being penetrated by a sharp explorer using light probing pressure (similar to the pressure used during probing depth measurements). Then, ozone gas (concentration of 2350 ppm and a flow rate of 615 cc/min) was applied via the healOzone X4 machine (Curozone, Germany) for 20 s to disinfect the remaining leathery dentine caries. Ozone gas was delivered to the carious lesions using disposable silicone cups purchased from the manufacturer, and used to secure an adequate seal to prevent any ozone leakage. After that, the pulpal floor and adjacent wall of each cavity were covered by a glass ionomer cement (Riva Self Cure High Viscosity, SDI, Australia) followed by a bonded composite resin restoration (Filtek Z250 Universal Restorative, 3 M ESPE, USA; Single Bond Universal Adhesive, 3 M ESPE, Germany; Ultra-Etch 35% phosphoric acid, Ultradent, USA).

In contrast, carious lesions in group 2 were treated traditionally by a total removal of caries under copious water irrigation using diamond burs (Edenta, Switzerland) mounted on a contra-angled high speed handpiece (Coxo CX207-B, COXO Medical Instrument Co., China) and then with tungsten carbide burs mounted on a slow speed handpiece (Gold-U-CA, AppleDental, China) before placement of the glass ionomer cement (Riva Self Cure High Viscosity, SDI Australia) followed by composite restoration (Filtek Z250 Universal Restorative, 3 M ESPE, USA; Single Bond Universal Adhesive, 3 M ESPE, Germany; Ultra-Etch 35% phosphoric acid, Ultradent, USA).

After restoration, the participants were instructed to attend 24 h later for review and further assessment of pain. Participants’ tooth pain was assessed using the same VAS score.

The restored teeth in both groups were assessed on a weekly basis for 1 month then each month for 6 months. Then, they were assessed every 6 months till the end of the study. On each recall visit, the teeth were assessed for vitality, tenderness to percussion, swelling or change in colour. Assessment for the need for endodontic treatment was carried out by evaluation of tooth vitality using cold ice sticks and comparison to other teeth. The participants were instructed to report to the clinic if they feel any symptoms related to the restored tooth such as swelling or spontaneous pain that woke the participant up or keep them awake at night.

In this study, tooth restoration was carried out by one investigator (M.K.AL-O), meanwhile pain assessment and need for endodontic treatment was assessed via another investigator (R.A.H) who was blinded to the performed restoration protocol. Intra-examiner reliability was verified through evaluating 15 duplicate pain assessments by the same investigator, and Kappa was found to be adequate (κ = 0.92).

### Statistical analysis

Data analysis was performed using the SPSS computer software (Statistical Package for the Social Sciences, v19.0; IBM, Armonk, NY, USA). The Kolmogorov–Smirnov test was used to test normal distribution of the data. The Spearman correlation test was utilized to explore the relationships between age on one side and the pain scores at baseline, pain scores 24 h after restoration, and the need for RCT. The Fisher’s Exact test was used to explore the relationship between the need for RCT and pain scores. The Mann Whitney test was used for comparisons between males and females within each group. The Mann Whitney test was also used for comparisons between ozone and conventionally treated groups. The statistical significance for this study was set at probability values of P ≤ 0.05 and a 95% confidence interval.

The hierarchical multiple regression analysis considering the confounding effects of gender, age, and treated tooth was utilized to identify the contribution of the treatment protocol towards the VAS pain scores after 24 h of treatment. Also, the hierarchical multiple logistic regression analysis considering the confounding effects of gender, age, and treated tooth was utilized to identify the contribution of the treatment protocol towards the need for RCT throughout the study period.

For this experiment, the sample size was calculated via the G*Power 3.1.9.7 software (Heinrich-Heine-University, Düsseldorf, Germany). The minimum sample size was estimated to be 34 participants per group utilizing a priori power analysis with a significance level of 5%, a statistical power of 80%, and an effect size of 20%. Therefore, 42 participants per group were recruited into this experiment to compensate for any possible dropouts.

## Results

The study population consisted of 84 participants (42 females and 42 males). The data from all included participants was analyzed. None of the participants were lost to follow up (drop out ratio was zero %). Participants age ranged between 20 and 27 years old (mean age = 23.92 years old, SD =  ± 2.01 years, SE =  ± 0.22 years).

The mean pain visual analogue scale score for participants with the traditional treatment was 7.98 (SD ± 0.78, 95% CI = 7.73–8.22, SE = 0.120) before treatment and 5.40 (SD ± 1.62, 95% CI = 4.90–5.91, SE = 0.251) after 24 h whilst these were 7.76 (SD ± 0.76, 95% CI = 7.53–8.00, SE = 0.117) and 0.52 (SD ± 0.99, 95% CI = 0.21–0.83, SE = 0.153) for the ozone treated participants. After 2 months, 8 (19.0%) participants required root canal treatment from the traditional management group whilst 1 (2.4%) participant required root canal treatment in the ozone group. Gender distributions of pain scores and the need for RCT are presented in Table 1.

**Table 1:** Differences between genders regarding baseline pain scores, pain scores after 24 h of treatment, and the need for root canal treatment within each study group.

Variables	Ozone group		Traditional group		Mann Whitney test		
	Males	Females	Males	Females	MW-U	Z	P
**Tooth pain at baseline**					163.0	− 1.562	.118
Mean	7.57	7.95	8.05	7.90			
SD	0.746	0.740	0.740	0.831			
CI	7.23–7.91	7.62–8.29	7.71–8.38	7.53–8.28			
SE	0.163	0.161	0.161	0.181			
**Tooth pain after 24 h**							
Mean	0.33	0.71	5.57	5.24	185.5	− 1.028	.304
SD	0.483	1.309	1.599	1.670			
CI	0.11–0.55	0.12–1.31	4.84–6.30	4.48–6.00			
SE	0.105	0.286	0.349	0.365			
**Need for RCT**							
Frequency	0	1	4	4	210.0	− 1.000	.317

MW-U = Mann Whitney U test coefficient, Z = Z statistic, P = two tailed probability, SD = Standard deviation, CI = 95% Confidence intervals of the mean, SE = Standard error of the mean, RCT = Root canal treatment.

After 2 years follow up, 8 participants required root canal treatment from the traditional management group whilst 1 participant required root canal treatment in the ozone group. Among the traditional group, one participant required root canal treatment 3 days after filling, 2 participants after one week, 3 participants after two weeks, and 2 participants after four weeks following restoration. However, only one participant required root canal treatment after 1 week among the ozone group.

In the ozone group, age was not related to pain scores before treatment (Spearman correlation = -0.135, p = 0.394), pain scores after 24 h (Spearman correlation = -0.065, p = 0.685), and the need for RCT (Spearman correlation = -0.046, p = 0.774). Also in the traditional group, age was not related to pain scores before treatment (Spearman correlation = 0.158, p = 0.319), pain scores after 24 h (Spearman correlation = 0.001, p = 0.996), and the need for RCT (Spearman correlation = -0.041, p = 0.798).

In both groups, it was found that gender had no significant relationship with pain scores at baseline, pain scores 24 h after restoration, or the need for RCT (p > 0.05) (Table 1).

The need for RCT was not related to the pain scores at study baseline before ozone (Fisher’s Exact test value = 5.494, p = 0.190) as well as traditional intervention (Fisher’s Exact test value = 4.786, p = 0.091). On the other hand, the need for RCT was significantly related to pain scores 24 h after restoration among the ozone group (Fisher’s Exact test value = 9.069, p = 0.024) as well as traditional treatment group (Fisher’s Exact test value = 27.537, p < 0.0001).

Mann Whitney test showed significant differences between groups in terms of VAS scores of pain after 24 h of treatment (p < 0.0001) and the need for RCT after 2 years follow up (p = 0.014) (Table 2). Ozone treatment was associated with less pain and less need for RCT.

**Table 2:** Differences of dental pain and the need for root canal treatment between ozone and traditional treatment groups (n = 84, 42 participants for each group).

Statistics	Tooth pain at baseline	Tooth pain after 24 h	Need for RCT
MW-U	758.0	28.5	735.0
Z	− 1.187	− 7.787	− 2.455
P	.235	< .0001	.014

MW-U = Mann Whitney U test coefficient, Z = Z statistic, P = Two tailed probability, RCT = Root canal treatment.

The hierarchical multiple regression analysis considering the confounding effects of gender, age, and treated tooth revealed that the treatment protocol has significant contribution towards the VAS pain scores after 24 h of treatment (p < 0.0001) (Table 3). Also, the treatment protocol has significant contribution towards the need for RCT after 2 years follow up (p = 0.038) (Table 4).

**Table 3:** Hierarchical multiple regression analysis to predict the contribution of the treatment protocol towards the VAS pain scores after 24 h of treatment (n = 84).

Model*	Predictors	Unstand Co		Stand Co	t	Sig	95% CI for B	
		B	SE	Beta			Lower bound	Upper bound
Model 1 R^2^ = .008	(Constant)	4.753	3.930	–	1.209	.230	− 3.068	12.574
	Gender	.087	.624	.016	.140	.889	− 1.155	1.329
	Age	− .094	.157	− .067	− .597	.552	− .405	.218
	Affected tooth	.133	.284	.053	.471	.639	− .431	.698
Model 2 R^2^ = .771	(Constant)	− 4.475	1.981	–	− 2.258	.027	− 8.418	− .531
	Gender	.035	.301	.006	.116	.908	− .565	.635
	Age	− .002	.076	− .002	− .030	.976	− .153	.149
	Affected tooth	.054	.137	.021	.393	.695	− .219	.327
	Treatment protocol	4.875	.300	.877	16.246	< .0001	4.278	5.472

R^2^ = Coefficient of determination, Unstand Co = Unstandardized coefficient, Stand Co = Standardized coefficient, B = Beta statistics, SE = Standard Error, t = t statistics, Sig = Significance of probability (P value), CI = Confidence intervals.

*Significance of F statistic change was P = .886 and P < .0001 for Model 1 and Model 2 respectively.

**Table 4:** Prediction of the contribution of treatment protocol towards the need for root canal treatment among the study population using the hierarchical logistic regression analysis (n = 84).

Variable blocks*	B	SE	df	Sig	Exp (B)	Exp (B) 95% CI	
						Lower	Upper
**Block 1 (Nagelkerke R**^**2**^** = .011. Hosmer and Lemeshow test probability value (P) = .594)**							
Gender	.313	.727	1	.667	1.368	.329	5.686
Age	− .041	.179	1	.819	.960	.675	1.364
Affected tooth	.170	.325	1	.601	1.185	.627	2.240
Constant	− 2.035	4.388	1	.643	.131	–	–
**Block 2 (Nagelkerke R**^**2**^** = .166. Hosmer and Lemeshow test probability value (P) =.385)**							
Gender	.312	.751	1	.678	1.366	.314	5.948
Age	− .019	.198	1	.925	.982	.665	1.448
Affected tooth	.158	.343	1	.646	1.171	.598	2.293
Treatment protocol	2.257	1.088	1	.038	9.551	1.133	80.507
Constant	− 6.383	5.241	1	.223	.002	–	–

B = the B coefficient of the model, SE = Standard error, df = Degree of freedom, Sig. = Significance of two tailed probability value (P), Exp (B) = Exponentiated B coefficients, CI = Confidence intervals. *The predicted overall percentage for each Block equals 89.3%.

## Discussion

The outcomes of this study showed that in symptomatic deep carious lesions approaching the pulp, 20 s of ozone application on partially removed deep caries before a restoration would lead to less pain levels and less need for endodontic treatment in comparison to a conventional treatment protocol of total caries removal and a restoration. Therefore, the null hypothesis was rejected.

In this experiment, the healozone machine was utilized to supply ozone because it has been shown to be safe since its ozone releasing system is effectively sealed before the machine generates ozone^56,57^. Ozone gas is delivered to the tooth using different sizes of disposable silicone cups supplied by the manufacturer and these guarantee a perfect seal to avoid ozone leakage. Each cup is pressed against the tooth in order to ensure that the maximum ozone is delivered directly into the tooth. Furthermore, application of ozone via the healozone machine is better than many other ozone or other pharmaceutical application techniques as it only functions locally and not on large areas like mouth washes, and therefore does not negatively impact on the oral cavities resident protective microflora.

The outcomes of this experiment showed that pain levels after 24 h following restoration were less within the ozone treated group. This could be partly due to the known analgesic characteristics of ozone^45,47,51–53,58,59^.

Ozone has been found to decrease pain^30,45,47,51–53,60–62^, decrease tooth sensitivity^51–53,60,61^, and boost regeneration and improve healing^30,45–47,60–62^.

Also, previous studies showed that ozone reduces pain caused by lumbar disc herniation^63^. It also possesses strong anti-inflammatory and anti-oxidant effects against oxidative stress-induced tissue injuries^50^. Furthermore, ozone enhances the activity of antioxidant enzymes (including glutathione peroxidase, superoxide dismutase, and catalase) which combat inflammation^30,47,50^. In addition, it restrains inflammatory pathways by reducing prostaglandin production and inactivating cyclooxygenase^54^.

Reduced tooth pain after ozone application might also be attributed to reduction in number and diameter of open dentinal tubules^64,65^ and collagen degradation^66^ which might potentially block the dentinal tubules mechanically.

This is in agreement with the results of previous studies that reported less pain when ozone was used for other dental and oral applications such as treatment of recurrent oral ulcers, treatment of denture induced ulcers, extraction of third molars, management of jaw osteonecrosis, dentine hypersensitivity treatment, and bleaching^45,46,51–53,67–69^.

Besides, the findings of this experiment demonstrated that ozone treatment of deep caries was associated with less need for RCT than conventional treatment. This could also be attributed to the fact that ozone is effective for killing cariogenic microorganisms^1–5,8,12–27,29^.

Also, ozone prevents formation of bacterial biofilms on dentine by changing dentine wettability through oxidation of organic elements (such as proteins, carbohydrates, glycolipids and acids)^8,12,17,30,31^.

Nevertheless, ozone has limits to the distance it can infiltrate into a carious lesion. Previous researchers found that ozone did not decrease bacterial count in very deep large carious lesion or deep non cavitated lesion^2,5^. This indicates that direct contact between ozone and caries is required to achieve the desired outcomes as the ozone needs to penetrate the lesion to be effective.

For that reason, direct ozone application into open cavities following partial caries removal leaving only about one mm of caries was the tested treatment protocol in this experiment.

Also, ozone has beneficial effects on the pulp and can neutralize toxic endotoxins that irritate the pulp, and thus help the pulp to recover. As discussed before, ozone has strong anti-inflammatory and anti-oxidant properties against oxidative stress-induced tissue injuries^47,50^. Moreover, ozone boosts the activity of antioxidant enzymes (including glutathione peroxidase, superoxide dismutase, and catalase) which counteract inflammation^30,47,50^. Additionally, it controls inflammatory pathways by decreasing prostaglandin release and inactivating cyclooxygenase^54^.

The above discussion corroborate the anti-inflammatory, anti-oxidative and analgesic merits of ozone for dental pulp tissues^49^, and this would explain the efficiency of ozone in boosting healing and avoiding injury of pulp tissues.

This is in agreement with previous findings that ozone application after partial removal of caries from deep carious lesions in deciduous teeth would avoid the need for pulpotomy and result in success rates similar to pulpotomy^34^.

Also, other researchers found that ozone treatment either alone or combined with a re-mineralizing agent was efficient for remineralization of shallow as well as initial pit, fissure, root and proximal carious lesions in permanent and deciduous teeth^4,6,7,9,25,26,28,32,33^. The oxidative capacity of ozone allow destruction of the substances that demineralise dental tissues and prevent remineralization (such as formic, lactic, and pyruvic organic acids)^1,29^. Ozone was also found to reduce the number of microorganisms in deep carious lesions, and was associated with drier and harder dentine consistency^70–72^.

With the current findings in mind, it is useful to use ozone for management of deep caries as this would potentially reduce pain and the need for RCT, hence potentially improve patients’ compliance with caries treatment. If one is to leave some deep caries in an attempt not to expose the pulp, it would certainly seem more logical to aim to kill the acidogenic and aciduric microorganisms in the deep caries before placing any restoration. In addition, ozone treatment of deep caries might allow a less invasive approach for caries management that permits treatment of the cause of dental caries and help dental professionals to avoid root canal treatment in many cases.

Previous reports concluded that using ozone for management of caries was also associated with less patient anxiety^28,73^. Furthermore, a side gain of ozone application is that it does enhance the colour of teeth^51–53,74–77^. After all, ozone is adequately controlled via the dentist since the healozone machine permits adequate control of delivery site, concentration, volume and flow rates of ozone. Moreover, ozone application is rapid, convenient, less costly, and not irritant to dental tissues.

A limitation of this study was that the current experiment tested immediate and short-term effects of ozone application on deep carious lesions.

Future long term investigations are urged on larger samples to evaluate the effects of ozone for management of deep carious lesions approaching the pulp.

Furthermore, studies are requested to investigate the potential effectiveness of ozone in treatment of deep caries in comparison to other deep caries management techniques. Also, future studies are required to compare ozone and SDF for management of caries. In addition, future studies are needed to explore dentists’ views and patients’ compliance and satisfaction with using ozone for deep caries management in comparison to other techniques.

Further studies are also requested to achieve definite conclusions concerning whether adequate cavity sealing would result in death of viable bacteria left beneath tooth filling. Moreover, to discover whether it is required to disinfect dentine before restoration and which are the best techniques to disinfect residual dentine. Some researchers reported that leaving infected dentine and simply sealing cavity with restoration was a successful treatment paradigm for deep caries^77–80^.

However, other researchers have demonstrated caries progression when caries was sealed in without disinfection^81–83^. In order to resolve this controversy, a future study is planned with 2 groups, leaving about 1 mm of caries in each; one using ozone to disinfect dentine and the other not using ozone and simply sealing in the caries.

## Conclusion

This study concluded that ozone treatment of symptomatic teeth with deep, carious lesions almost reaching the pulp shows promise for a more conservative approach to treat deep caries as well as being associated with less postoperative pain and less need for root canal treatment compared to a traditional method.

## Data Availability

Data generated and analysed during this study are available from the corresponding author upon request to the following email: alomirim@yahoo.co.uk.
